# A new *fast* real‐time PCR method for the identification of three sibling *Apodemus* species (*A. sylvaticus*,* A. flavicollis*, and *A. alpicola*) in Italy

**DOI:** 10.1002/ece3.4026

**Published:** 2018-04-17

**Authors:** Giulia Sozio, Valentina Curini, Ilaria Pascucci, Cesare Cammà, Marco Di Domenico

**Affiliations:** ^1^ Istituto Zooprofilattico Sperimentale dell'Abruzzo e del Molise “G. Caporale” Teramo Italy

**Keywords:** alpine field mouse, cytochrome B, probe, TaqMan, wood mouse, yellow‐necked mouse

## Abstract

The identification of field mice *Apodemus flavicollis*,* Apodemus sylvaticus,* and *Apodemus alpicola* represents a challenge for field scientists due to their highly overlapping morphological traits and habitats. Here, we propose a new *fast* real‐time PCR method to discriminate the three species by species‐specific TaqMan assays. Primers and probes were designed based on the alignment of 54 *cyt‐b* partial sequences from 25 different European countries retrieved from GenBank. TaqMan assays were then tested on 133 samples from three different areas of Italy. Real‐time PCR analysis showed 92 samples classified as *A. flavicollis*, 13 as *A. sylvaticus,* and 28 as *A. alpicola*. We did not observe any double amplification and DNA sequencing confirmed species assignment obtained by the TaqMan assays. The method is implementable on different matrices (ear tissues, tail, and blood). It can be used on dead specimens or on alive animals with minimally invasive sampling, and given the high sensitivity, the assay may be also suitable for degraded or low‐DNA samples. The method proved to work well to discriminate between the species analyzed. Furthermore, it gives clear results (amplified or not) and it does not require any postamplification handling of PCR product, reducing the time needed for the analyses and the risk of carryover contamination. It therefore represents a valuable tool for field ecologists, conservationists, and epidemiologists.

## 1 INTRODUCTION

*Apodemus* is a genus of field mice distributed in the Paleartic Region including 21 species and four groups: *Sylvaemus*,* Apodemus*,* Gurkha,* and *Argenteus* (Musser & Carleton, 2005). Seven species occur in Europe with more or less overlapping ranges. Many of these species are targeted by ecological, conservation, and epidemiological studies that have to face the need of distinguishing between co‐occurring species, especially when they are similar.

In most Europe, in particular, the identification of yellow‐necked mouse (*Apodemus flavicollis*) and wood mouse (*Apodemus sylvaticus*), both belonging to *Sylvaemus* group, represents a challenge for field scientists due to their highly overlapping morphological traits, habitats, and geographic range.

*Apodemus sylvaticus* is distributed throughout Europe (excluding Finland and northern parts of Scandinavia, the Baltics and Russia) and in some regions of North Africa (Schlitter et al., 2016). *Apodemus flavicollis* has a very similar distribution that extends northwards in southern Finland, in the Baltics, western Russia and some regions of Anatoly (whereas it is absent in Africa and Iceland) (Amori et al., 2016).

The physical resemblance of these two species is particularly marked in the southern part of their range, where discrimination based on the sole external characters such as body size and pelage color has proven to be unfeasible (Filippucci, Cristaldi, Tizi, & Contoli, 1984; Montgomery, 1980; Niethammer, 1978). Even though the two species have different ecologies, with *A. flavicollis* being more strictly associated to forested habitats and *A. sylvaticus* being also found in forest‐edges, ecotones and in association with agricultural and anthropized environments, their ecological preferences partially overlap and the two species often live in syntopy (Marsh & Harris, 2000; Mitchell‐Jones et al., 1999). The discrimination between these two siblings, albeit distinct species, is therefore very important for ecological, epidemiological, evolutionary, and B‐chromosome studies. The situation is even more complex in the Alpine region, where a third very similar species, the Alpine field mouse (*Apodemus alpicola*) occurs in sympatry and often in syntopy with the other two. The Alpine field mouse is distributed throughout the Alps (France, Switzerland, Germany, Italy, and Austria).

To date, different approaches have been developed and discussed to tackle the need of discriminating between *Apodemus* species, but the debate is still far from being solved. Cranial measurements have proven to be effective in many cases (e.g., Barčiová & Macholán, 2009; Debernardi et al.^2003^; Jojić, Bugarski‐Stanojević, Blagojević, & Vujošević, 2014; Reutter, Hausser, & Vogel, 1999), but their validity tends to be population‐specific. Furthermore, they can only be used on dead animals or rests of them (e.g., on skulls found in owl pellets). External morphological measures taken on dead or alive individuals, such as pelage color pattern, body size, length of tail, hear, hind foot, have also been used to identify species (e.g., Debernardi et al. 2003; Filippucci et al., 1984; Kuncová & Frynta, 2009; Niethammer, 1978), even though a high degree of uncertainty remains for individuals with overlapping characters (Bartolommei et al. 2016). Recently, an innovative bioacoustic approach, not needing animal sacrifice and carried out on awake animals, has been developed to help the classification of individuals based on distress calls emitted during handling (Ancillotto et al., 2017).

All the aforementioned methods, however, cannot provide a 100% classification success, as cranial, external, and bioacoustic traits present continuous gradients and animals with intermediate characters (including juveniles and sub‐adults) cannot be classified correctly.

To date, the only classification techniques for *Apodemus* spp. known to provide high rates of success rely on cytogenetic or molecular methods. Although the three species have similar karyotypes, Q/C banding has proved to be effective as a discrimination method (Engel et al., 1973; Hirning, Schulz, Just, Adolph, & Vogel, 1989), but it requires animal sacrifice. Molecular methods, including protein electrophoresis (Filippucci, 1992; Filippucci, Macholan, & Michaux, 2002; Orlov, Bulatova, Nadjafova, & Kozlovsky, 1996; Vogel, Maddalena, Mabille, & Paquet, 1991) and DNA analysis (e.g., sequencing, PCR), can instead be applied on samples from alive animals (e.g., a small sample of ear or tail tissue). Michaux et al. (2001) described an assay based on a conventional PCR with species‐specific primers targeting a fragment of cytochrome b mitochondrial gene (*cyt‐b*). Given its simplicity and clarity of results (PCR product present or not), this method is one of the most commonly used in the literature, being preferred to other expensive methods such as DNA sequencing. However, in their recent work, Bugarski‐Stanojević, Blagojević, Adnađević, Jovanović, and Vujošević (2013) showed that the method by Michaux et al. (2001) is subject to a certain degree of misidentification, probably due to the low specificity of the chosen primers or to the existence of nuclear copies of mitochondrial genes (pseudogenes) which determine false‐positive results (Dubey, Michaux, Brunner, Hutterer, & Vogel, 2009). In their work, Bugarski‐Stanojević et al. (2013) compared the method with two alternative molecular assays with higher specificity: the arbitrarily primed‐PCR (AP‐PCR) and the intersimple sequence repeat‐PCR (ISSR‐PCR). Both methods result in species‐specific DNA profiles that can be visualized through gel electrophoresis. Such methods, however, referring to arbitrary sequence PCR, generally exhibit poor interlaboratory reproducibility that hinders their widespread use. Moreover, postamplification handling of PCR products is required, increasing the time needed for the analyses and introducing the risk of carryover contamination.

Here, we propose a new *fast* real‐time PCR method to distinguish between the three species. Real‐time PCR using TaqMan probes has been reported by different authors as a fast and sensitive method for the identification of species (Cammà, Di Domenico, & Monaco, 2012; Di Domenico, Di Giuseppe, Wicochea Rodríguez, & Cammà, 2017; Overdyk, Braid, Naaum, Crawford, & Hanner, 2016). It does not require any postamplification step and can be easily automated allowing the analysis of large numbers of samples. Moreover, the application of specific primers in combination with fluorogenic probes considerably increases reaction specificity.

## 2 MATERIALS AND METHODS

### 2.1 Design of primers and probes

A total of 54 *cyt‐b* partial sequences of *A. flavicollis*,* A. sylvaticus,* and *A. alpicola* from 25 different countries were retrieved from GenBank (Accession numbers in Table 1). Sequences were aligned with software MegAlign (DNASTAR Lasergene 10) and species‐specific primers and TaqMan probes were designed based on differences between species (Table 2). Primer Express Software 3.0.1 (Applied Biosystems) was also used to exclude the presence of secondary structures between primers and probes that would reduce reaction efficiency.

**Table 1:** Geographic origin, references and GenBank accession numbers of *Apodemus cyt‐b* sequences used to design primers and probes for real‐time PCR assays

Species	Geographic origin		Accession number	Reference
*A. flavicollis*	Belgium	Gembes	AJ298601	Michaux et al. (2001)
	France	Allier	AJ311151	Michaux, Chevret, Filippucci, and Macholan (2002)
		Allier	AJ298602	Michaux et al. (2001)
	Germany	Bielefeld	AJ298603	Michaux et al. (2001)
		Konstanz	AF159392	Martin, Gerlach, Schlötterer, and Meyer (2000)
	Greece	Mt. Olympus	AJ631968	Michaux, Libois, and Filippucci (2005)
		/	JF819967	Krystufek, Luznik, and Buzan (2012)
		Peloponnese	AJ605625	Michaux, Libois, Paradis, and Filippucci (2004)
	Italy	Abruzzes	AJ311150	Michaux et al. (2002)
		Aspromonte	AJ298604	Michaux et al. (2001)
		Grosseto	AJ605635	Michaux et al. (2004)
	Spain	Navarra	AJ631969	Michaux et al. (2005)
	Sweden	Gotland	AJ631970	Michaux et al. (2005)
	Switzerland	Champéry	AB032853	Serizawa, Suzuki, and Tsuchiya (2000)
	Ukraine	Chernobyl	AF127539	Makova, Nekrutenko, and Baker (2000)
	Bosnia and Herzegovina	/	JF819970	Krystufek et al. (2012)
	Czech Republic	Karsperske	AJ605609	Michaux et al. (2004)
	Hungary	Debrecen	AJ605634	Michaux et al. (2004)
	Macedonia	Bistria	AJ605644	Michaux et al. (2004)
	Romania	Cheile garlistei	AJ605647	Michaux et al. (2004)
	Russia	Voronezh	AJ605654	Michaux et al. (2004)
		Volgograd	AJ605652	Michaux et al. (2004)
	Slovenia	Asan cesma	AJ605657	Michaux et al. (2004)
*A. sylvaticus*	Belgium	Ardennes	AJ298605	Michaux et al. (2001)
		Ardennes	AJ298598	Michaux et al. (2001)
	France	Eastern Pyrenees	AJ298599	Michaux et al. (2001)
		Eastern Pyrenees	AJ311149	Michaux et al. (2002)
	Italy	Aspromonte	AJ511923	Michaux, Magnanou, Paradis, Nieberding, and Libois (2003)
		Aspromonte	AJ511924	Michaux et al. (2003)
		Latium	AJ298600	Michaux et al. (2001)
		Latium	AJ311148	Michaux et al. (2002)
	The Netherlands	/	AB033695	Suzuki, Tsuchiya, and Takezaki (2000)
	Ukraine	Chernobyl	AF127536	Makova et al. (2000)
		Chernobyl	AF127537	Makova et al. (2000)
		Chernobyl	AF127538	Makova et al. (2000)
	United Kingdom	Frenchay	AF127543	Makova et al. (2000)
	Poland	Pomorze	KX159689	Herman et al. (2016)
	Ireland	Galway	KX159658	Herman et al. (2016)
	Ireland	Wexford	KX159669	Herman et al. (2016)
	Portugal	Vila Real	KX159696	Herman et al. (2016)
	Portugal	Serra da Estrela	KX159697	Herman et al. (2016)
	Luxembourg	/	KX159672	Herman et al. (2016)
	Norway	Rogaland	KX159647	Herman et al. (2016)
	United Kingdom	York	KX159644	Herman et al. (2016)
	United Kingdom	Warwick	KX159637	Herman et al. (2016)
	Iceland	Shetland	KX159653	Herman et al. (2016)
*A. alpicola*	Austria	Klosterle	AY179495	Reutter et al. (2003)
		Stuben	AB032854	Serizawa et al. (2000)
		Stuben	AY179496	Reutter et al. (2003)
		Vorarlberg	AJ311153	Michaux et al. (2002)
	France	Mt. Cenis	AY179497	Reutter et al. (2003)
		Savoie	AJ311152	Michaux et al. (2002)
	Switzerland	Sanetsch	AY179494	Reutter et al. (2003)
		/	AF159391	Martin et al. (2000)

**Table 2:** Primers and TaqMan probes designed for the identification of *Apodemus* species. Amplicon length and optimized concentrations of primers and probes are also reported

Oligo	Sequence (5′→3′)	Concentration (nmol/L)	Amplicon length (bp)
*Apodemus flavicollis*			
Forward	GCCGAGACGTAAATTATGGATGAT	150	
Reverse	TCCTACGTGTAGAAATAAGCAAATGAA	150	89
Probe	FAM‐AATTCGATATTTACACGCAAACGGAGCCTC‐TAMRA	150	
*Apodemus sylvaticus*			
Forward	ATCATGATGAAACTTCGGCTCAT	200	
Reverse	AGTCAGCCATAATTTACGTCTCGAC	200	150
Probe	JOE‐ATCCAAATCCTCACAGGCTTATTTCTAGCAATACA‐TAMRA	200	
*Apodemus alpicola*			
Forward	AATCAAAGACATTCTAGGAGTACTCATAATAATC	600	
Reverse	AGTATTTAGTGGGTTGGCAGGC	600	118
Probe	FAM‐TCATTCCTTATAATACTAGTACTCTTCTTCCCAGACCTTC‐TAMRA	250	

### 2.2 Sensitivity, specificity and repeatability of the real‐time PCR assays

Three replicates of five tenfold DNA serial dilutions from 20 ng/ml to 2 pg/ml of each species were prepared to create the standard curve. The efficiency (*E*) of the real‐time PCR was calculated according to the formula *E* = (10^−1/slope^ − 1) × 100 (Vaerman, Saussoy, & Ingargiola, 2004). The lowest dilution in the linear dynamic range producing all positive results was considered to assess the limit of detection (LOD). The repeatability of the methods was estimated calculating the coefficient of variation (CV) relative to the analysis of twenty‐four replicates, in three different runs for the different LOD DNA concentration relative to the target species *A. sylvaticus*,* A. flavicollis,* and *A. alpicola*. The analytical specificity was evaluated using the three target species as input DNA and DNA from human, *Mus musculus, Myodes glareolus*, and *Rattus norvegicus* as negative controls. Specificity was also evaluated in silico by comparing the designed PCR primers and probes with the sequences, deposited in NCBI public database, of other similar *Apodemus* species (*A. uralensis* and *A. witherbyi*, of the *Sylvaemus* group, sharing part of the distributional range with the target species).

### 2.3 Samples and DNA extraction

**Figure 1:**
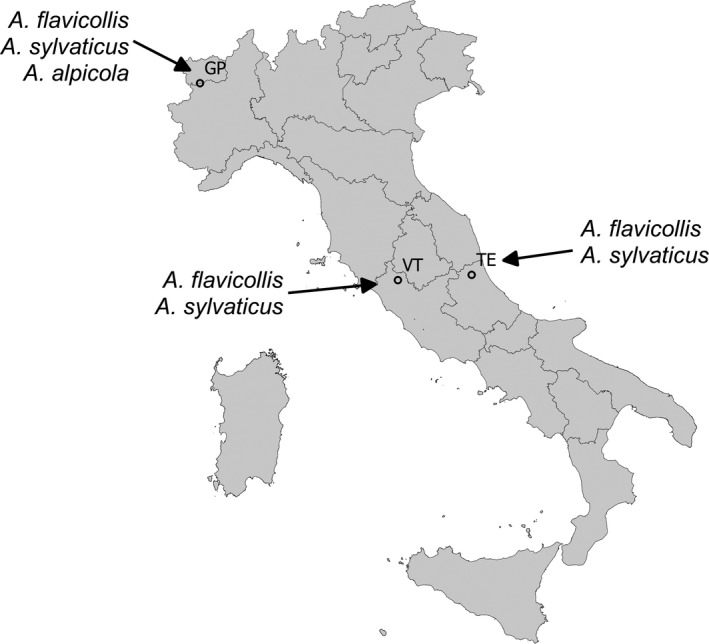
Geographic origin of the 133 Italian *Apodemus* samples tested with real‐time PCR for species discrimination. GP, Gran Paradiso National Park; VT, Viterbo area; TE, Teramo area

We collected a total of 133 samples from three different areas of Italy to ensure a high genetic variability between samples. Two areas, where *A. flavicollis* and *A. sylvaticus* occur in sympatry, are located in Central Italy and are separated by the Apennine chain (TE: Teramo and VT: Viterbo; Figure 1). The third area is located in Northern Italy (GP: Gran Paradiso National Park; Figure 1), were the third species, *A. alpicola*, is sympatric with the other two. Samples from VT (*N* = 11) were provided as DNA extracts stored at −20°C (previously obtained with the salting‐out protocol described by Aljanabi & Martinez, 1997 from ear tissues). Samples from TE (*N* = 85) were blood samples added with 10 μl sodium citrate (5%) and stored at +4°C before DNA extraction, while samples from GP (*N* = 37) were provided as ear/tail tissue samples stored in 70% ethanol. Samples from TE and GP were extracted with Maxwell 16 System instrument and Maxwell 16 Blood or Tissue DNA kits (Promega), according to producer's protocols. DNA concentration of all samples was measured with NanoDrop 1000 V3.8.1 (Thermo Fisher Scientific) and then diluted up to 2 ng/μl.

### 2.4 Real‐time PCR

Real‐time PCR reactions were carried out in 20 μl volumes including 10 μl of TaqMan Fast Universal PCR Master Mix 2× (Applied Biosystems) and 5 μl of 2 ng/μl DNA. The optimized concentration of primers and probes is reported in Table 2. A duplex assay, with the use of two different fluorescent markers, was optimized for *A. flavicollis* and *A. sylvaticus* (FAM and JOE, respectively). A simplex assay (FAM) was optimized for *A. alpicola*. Reactions were performed in a 7900HT Fast real‐time PCR System (Applied Biosystems) with the following thermal profile: 20 s at 95°C followed by 40 cycles of 1 s at 95°C and 20 s at 60°C.

### 2.5 DNA sequencing

A subsample of 33 DNA extracts (11 for each species) was sequenced to confirm the real‐time PCR results. Samples were amplified through PCR with primer pair CB‐AF/CB‐AR2 (Reutter, Petit, Brünner, & Vogel, 2003), targeting an 866 bp fragment of *cyt‐b*. Reactions were carried out in 50 μl volumes with 0.03 U/μl AmpliTaq Gold (Applied Biosystems), 1 ×  PCR buffer (Applied Biosystems), 2.5 mmol/L MgCl_2_ solution (Applied Biosystems), 200 μmol/L dNTPs, 600 nmol/L primers, and 5 μl of DNA extract. Amplifications were carried out in a GeneAmp PCR System 9700 (Applied Biosystems) with the following thermal profile: 10 min at 95°C followed by 40 cycles of 30 s at 95°C, 30 s at 47°C and 30 s at 72°C plus 7 min at 72°C. PCR products were purified using the GeneAll ExpinTM PCR Kit (GeneAll) and sequenced with the BigDye Terminator v3.1 Cycle Sequencing Kit (Applied Biosystems) and the 3130XL Genetic Analyzer (Applied Biosystems) using the primers CB‐AF and CB‐AR2 in both directions. Sequences were assembled with SeqMan Pro (DNASTAR Lasergene 10). Based on sequence quality and overlapping of the two strands, we selected only the central part of the contig to be submitted on BLAST (Basic Local Alignment Search Tool) for species identification and to be deposited in GenBank. In the cases where we had poor‐quality sequences or found possible clues for pseudogenes (i.e., double peaks), we discarded the uncertain sequence, thus only indisputable sequences were included in the analyses.

## 3 RESULTS

### 3.1 Sensitivity, specificity, and repeatability of the real‐time PCR assays

The slope of the standard curve, the efficiency, and the correlation coefficient (*R* ^2^) of all three assays are reported in Table 3. The LOD calculated for the different species ranges between 10 and 100 pg as reported (Table 3). Moreover, the methods revealed a very high level of repeatability as assessed by the low values of the coefficient of variations as shown in the same Table 3.

**Table 3:** Detailed data of Sensitivity, Specificity and Repeatability relative to the three *Apodemus* species‐specific real‐time PCR assays

Species	Slope	Efficiency (*E*), %	*R* ^2^	LOD (pg)	CV
*Apodemus flavicollis* a	−3.50	90	.99	100	1.7
*Apodemus sylvaticus* a	−3.32	100	.99	10	0.8
*Apodemus alpicola*	−3.33	99	.99	10	0.5

Efficiency (*E*) was calculated with the formula *E* = (10^−1/slope^ − 1) × 100. Slope and coefficient of determination (*R* ^2^) were calculated by the standard curve. Limit of detection (LOD) was calculated on the latest DNA dilution giving all positives results. CV was calculated by the ratio between standard deviation and mean of *C*_t_ values at LOD DNA concentration.

a Data referred to the duplex real‐time PCR assay.

For each assay, the other two species were tested as nontarget DNA, using the same amount, and no cross‐amplifications were observed; human, *M. musculus*,* Myodes glareolus*, and *Rattus norvegicus* DNA did not produce fluorescent amplification signal in any of the three new developed assays.

In addition, in silico analyses confirmed the specificity also against other *Apodemus* species. In particular, primers and probe designed for the three different assays showed sequence identity against *A. whiterbyi* and *A. uralensis* lower than 92% even for the best records. An exception was *A. sylvaticus* probe against *A. uralensis *showing a 94% sequence identity; however, both forward and reverse primers mismatched in two nucleotides at the 3′ end, limiting the aspecific amplification of this nontarget species.

### 3.2 Real‐time PCR and DNA sequencing

Real‐time PCR reactions obtained a 100% success of amplification. Ninety‐two samples were classified as *A. flavicollis*, 13 as *A. sylvaticus* and 28 as *A. alpicola* (Table 3). We did not obtain any double amplification (Table 3).

After sequencing, based on sequence quality and overlapping of the two strands, we selected a good central fragment of 677 bp (of 866 bp). For some samples, sequencing ended with poor‐quality sequences that could not be used for the analysis or deposited. Other samples showed some clues of pseudogenes (i.e., double peaks) and also these sequences were excluded. We selected 33 good sequences (about 25%) to be deposited in GenBank. All the 33 sequences were submitted on BLAST and confirmed the classifications obtained by TaqMan assays (Table 4). Accession numbers for deposited sequences are provided in the Data accessibility section.

**Table 4:** Results of real‐time TaqMan assays for species discrimination and confirmation by DNA sequencing

Area	Sample type	*N*	TaqMan assays			DNA Sequencing
			FLA	SYL	ALP	Confirmed/sequenced
TE	Blood	79	+	−	−	
	Blood	6	−	+	−	6/6
VT	Ear tissue	7	+	−	−	5/5
	Ear tissue	4	−	+	−	2/2
GP	Ear/tail tissue	6	+	−	−	6/6
	Ear/tail tissue	3	−	+	−	3/3
	Ear/tail tissue	28	−	−	+	11/11
Total		133	92	13	28	33/33

FLA = *A. flavicollis*, SYL = *A. sylvaticus*, ALP = *A. alpicola*.

## 4 DISCUSSION

In this work, we provided a new *fast* real‐time PCR method for the discrimination of three *Apodemus* species. It can be used on dead specimens or on alive animals with minimally invasive sampling, a characteristic often required by ecology or conservation studies. The three assays proved to be a useful tool on different matrices (tissues from ear, tail, and blood). Real‐time PCR is characterized by a much higher sensitivity compared to conventional PCR, being able to detect even very low copies of DNA (Angelone‐Alasaad et al., 2015). For this reason, this modern method is now commonly used to amplify small or degraded samples (Holt et al., 2016; Lee, McCord, & Buel, 2014) or for diagnostic purposes (Caraguel, Stryhn, Gagne′, Dohoo, & Hammell, 2011). Although we only tested our method on fresh or well‐preserved samples, given its sensitivity, it is likely to work on degraded (e.g., museum specimen) or low‐DNA samples obtained through noninvasive sampling, such as feces and hair (e.g., obtained through hair‐tubes; Kanthaswamy, Premasuthan, Ng, Satkoski, & Goyal, 2012). Indeed, despite the DNA concentration used was 2 ng/μl, the assays show LODs three logs lower (2 pg/μl) then the input loaded in the test. The use of TaqMan probes considerably increases reaction specificity compared to other traditional methods (Kuboniwa et al., 2004). Accordingly, our assays proved to work well to discriminate between the species analyzed as we did not obtain any double amplification even in the presence of pseudogenes, a possible source of misclassification (Dubey et al., 2009). In silico analyses also suggest designed primers and probes to be species‐specific also versus other nontarget *Apodemus* species (*A. uralensis* and *A. witherbyi*). Method efficacy is therefore comparable to or higher than other molecular assays such as those proposed by Bugarski‐Stanojević et al. (2013) or by Michaux et al. (2001) respectively, without the need of postamplification handling of PCR products, thus reducing the risk of cross‐contamination and the time needed for the analyses. The intra‐ and interlaboratory reproducibility and interpretation of results are also much easier, as real‐time PCR results in a yes/no outcome (amplified/not amplified) and in a numeric value of *C*_t_ depending on DNA concentration. Moreover, the possibility of working in duplex and the chance of automation further reduce the cost and time needed for the assay, especially for high number of samples.

We designed primers and probes based on sequences from several European regions to maximize the geographic applicability of the method. The assay proved to work on populations from three different areas of Italy separated by mountain chains. Although these reasons give us strong clues about the robustness of the method, we cannot totally exclude that local genetic variability from other regions of Europe not taken into account in this study could lead to a lower specificity of primers and probes and to possible cross‐amplification. Similarly, although the in silico analyses suggest species‐specificity also against other nontarget *Apodemus* species, we did not directly test them and we cannot exclude that a certain level of cross‐amplification could occur. In all these hypothetical cases, the comparison of *C*_t_ values (not possible with traditional methods relying on visual interpretation of electrophoresis band patterns) may be a useful approach to identify correct species assignment even with doubtful amplifications.

## CONFLICT OF INTEREST

None declared.

## DATA ACCESSIBILITY

DNA sequences: Genbank accession numbers KU975553‐KU975564.

## AUTHOR CONTRIBUTIONS

GS, CC, MDD, and IP conceived and designed the study; GS collected samples; GS, VC, and MDD performed analyses; GS and MDD wrote the manuscript; all the authors contributed to substantial manuscript improvement.
